# A Cohesin Subunit Variant Identified from a Peripheral Sclerocornea Pedigree

**DOI:** 10.1155/2019/8781524

**Published:** 2019-11-12

**Authors:** Bi Ning Zhang, Tommy Chung Yan Chan, Pancy Oi Sin Tam, Yu Liu, Chi Pui Pang, Vishal Jhanji, Li Jia Chen, Wai Kit Chu

**Affiliations:** ^1^Department of Ophthalmology & Visual Sciences, The Chinese University of Hong Kong, Hong Kong; ^2^Department of Ophthalmology, Hong Kong Sanatorium and Hospital, Hong Kong; ^3^Program in Systems Biology, Department of Biochemistry and Molecular Pharmacology, University of Massachusetts Medical School, 368 Plantation Street, Worcester, MA 01605, USA; ^4^Department of Ophthalmology, University of Pittsburgh, Pittsburgh, PA, USA; ^5^Shantou University/Chinese University of Hong Kong Joint Shantou International Eye Center, Shantou, China

## Abstract

**Background:**

Sclerocornea is a rare congenital disorder characterized with the opacification of the cornea. Here, we report a nonconsanguineous Chinese family with multiple peripheral sclerocornea patients spanning across three generations inherited in an autosomal dominant manner.

**Methods:**

This is a retrospective case series of a peripheral sclerocornea pedigree. Comprehensive ophthalmic examinations were conducted and assessed on 14 pedigree members. Whole-exome sequencing was used to identify the genetic alterations in the affected pedigree members. Lymphoblastoid cell lines (LCLs) were established using blood samples from the family members. Functional tests were performed with these cell lines.

**Results:**

Six affected and eight unaffected members of a family with peripheral sclerocornea were examined. All affected individuals showed features of scleralization over the peripheral cornea of both eyes. Mean horizontal and vertical corneal diameter were found significantly decreased in the affected members. Significant differences were also observed on the mean apex pachymetry between affected and unaffected subjects. These ophthalmic parameters did not resemble that of cornea plana. A *RAD21^C1348T^* variant was identified by whole-exome sequencing. Although this variant causes RAD21 R450C substitution at the separase cleavage site, cells from peripheral sclerocornea family members had no mitosis and ploidy defects.

**Conclusion:**

We report a family of peripheral sclerocornea with no association with cornea plana. A *RAD21* variant was found cosegregating with peripheral sclerocornea. Our results suggest that RAD21 functions, other than its cell cycle and chromosome segregation regulations, could underline the pathogenesis of peripheral sclerocornea.

## 1. Introduction

Sclerocornea is a rare corneal opacification disease. The transition between the sclera and cornea is indistinct in sclerocornea cases [1, 2]. Based on the affected area of the cornea and vision damages of the patients, there are two major types of sclerocornea: total and peripheral sclerocornea [1, 2]. Most of the total sclerocornea patients suffer complete blindness and their cases can be identified easily. However, peripheral sclerocornea patients still have useful vision, though limited, they are thus unwilling to seek medical advice, and rare cases of peripheral sclerocornea have been reported. Corneal transplantation is the only way to treat sclerocornea and restore vision in total sclerocornea patients. Given the shortage of cornea for transplantation and the high failure rate of keratoplasty for young children [3, 4], better understanding of the disease mechanism of sclerocornea is needed so that alternative treatments can be developed.

Cohesin comprises four core components: two structural maintenance of chromosomes subunits, SMC1 and SMC3, a kleisin subunit RAD21, and either one of the stromal antigens STAG1 or STAG2 [5–9]. Cohesin is a ring-structure protein complex that encircles sister chromatids and prevents premature sister chromatid separation. At centromeres and telomeres, before the onset of anaphase, removal of cohesin from chromosomes involves a separase cleavage pathway [10, 11]. The active separase cleaves RAD21 proteolytically after the arginine at positions 172 (Arg172) and 450 (Arg450) [12]. These cleavages on RAD21 open the cohesin complex to allow sister chromatid separation. Rad21 has been found to be highly expressed in both postnatal developing cornea and adult cornea in mice compared with another ocular tissue [13], suggesting potential roles of Rad21 in cornea development and cornea diseases.

In this study, we identified a Chinese family with multiple peripheral sclerocornea patients spanning three generations. Our genetic and genomic analyses discovered that the heterozygous variant at one of the separase cleavage sites of RAD21, R450C, is associated with peripheral sclerocornea. Although this variant led to reduced separase cleavage of RAD21, we did not observe cell cycle or ploidy alterations.

## 2. Materials and Methods

### 2.1. Patient Consent

This study adhered to the tenets of the Declaration of Helsinki and was approved by the institutional review board of Kowloon Central Cluster, Hospital Authority, Hong Kong (KC/KE-15-0223/ER-1). Consent forms were signed by participants from this peripheral sclerocornea pedigree.

### 2.2. Clinical Assessments

Visual acuity assessment, refraction, slit-lamp examination, intraocular pressure measurement by Goldmann Applanation Tonometer, and optic disc assessment were performed (Table 1). Axial lengths were determined with IOL Master V.5 (Carl Zeiss Meditec, Dublin, CA, USA). Corneal topography, pachymetry, anterior chamber depth, and anterior chamber angle were assessed using a swept-source OCT (CASIA SS-1000, Tomey, Japan). Retinal nerve fiber layer was evaluated with a spectral domain OCT (Cirrus HD-OCT, Carl Zeiss Meditec). Fasting-serum lipid profile was performed for all 14 members who attended the clinic. Patient I-1 underwent bilateral extracapsular cataract extraction for senile cataract treatment. Preoperative keratometry and refraction of patient I-1 were traced from patient records for analysis. Anterior chamber depth measurements of patient I-1 were excluded because of the pseudophakic status. In addition, one unaffected case (III-9) had received bilateral laser-assisted in situ keratomileusis (LASIK) refractive surgery. The keratometry, refraction, pachymetry, and intraocular pressure data of this case were excluded from statistical analysis.

### 2.3. Whole-Exome Sequencing (WES), Data Annotation, and Bioinformatic Analysis

WES analysis (Macrogen) was performed in four affected cases and two unaffected cases. Genomic DNA was extracted using QIAamp DNA Blood Mini Kit (Qiagen), and exome DNA was enriched with an Agilent SureSelect Human All Exon V5 kit (Agilent Technologies). Paired-end sequencing was performed on Illumina HiSeq 4000 (Supplementary Tables [Supplementary-material supplementary-material-1] and [Supplementary-material supplementary-material-1]).

To identify functional variants that were associated with sclerocornea, raw sequencing reads were mapped onto human reference genome (hg19) using Burrows-Wheeler Aligner (BWA) [14] (Supplementary [Supplementary-material supplementary-material-1]). Both SAMtools [15] and Genome Analysis Toolkit (GATK) [16] were used to call SNPs and indels. Around 77,000 variants were obtained for each case. Among all the variants, 28,593 variants that appeared in all the four affected members were filtered using an in-house pipeline to obtain 3,744 variants within protein coding regions. 2,838 variants were excluded since two unaffected members also carried them. Remaining variants were further filtered using the autosomal dominant disease model, the dbSNP135, and the 1000 Genomes datasets to exclude population associated variants. Functional impacts of the variants was predicted using SIFT [17] and PolyPhen2 [18]. Since sclerocornea is a rare disease, a minor allele frequency threshold at 0.0001 was applied to obtain 15 rare variants. Published data were used to further examine these 15 variants [19]. Five nonsynonymous SNPs were excluded since they appeared in a Han Chinese control group of 190 unrelated controls. Ten candidate variants were obtained and summarized in Supplementary [Supplementary-material supplementary-material-1].

### 2.4. Lymphoblastoid Cell Lines (LCLs)

Fresh blood was collected and lymphocytes were isolated using Ficoll-Paque (GE Healthcare). Isolated lymphocytes were transformed with B95-8 Epstein-Barr virus (ATCC) according to the described protocol [20]. 2 *μ*g/mL cyclosporin A (Sigma-Aldrich) was added to the culture medium until cells formed a rosette morphology.

### 2.5. Cell Culture

LCLs were cultured with Iscove's modified Dulbecco's medium (IMDM) supplemented with 20% fetal bovine serum (FBS) and antibiotics (100 U/mL penicillin and 100 *μ*g/mL streptomycin, Gibco) at 37°C in a 95% air-5% CO_2_ incubator.

### 2.6. LCL Characterization

Allele specific gene expression analysis was conducted with data from RNA sequencing (Macrogen). For cell cycle analysis, LCLs were fixed with 70% ethanol for 30 min at 4°C. Cells were then stained with 40 *μ*g/mL propidium iodide (Sigma-Aldrich) and 100 *μ*g/mL RNase A by incubating in dark at room temperature for 1 hr. Stained cells were analyzed by flow cytometry. RAD21 Western blot was conducted with anti-RAD21 antibody (1 : 750, ab42522, Abcam) and goat anti-rabbit IgG-HRP antibody (1 : 2000, sc-2004, Santa Cruz). GAPDH (1 : 2500, G9295, Sigma-Aldrich) was used as a loading control. For chromosome counting, 5 × 10^5^ LCLs were cultured in 2 mL medium and treated with 100 ng/mL colcemid (KaryoMAX, Gibco) for 1 hr. Cells were incubated with 50 mM KCl at 37°C for 15 min. Cells were fixed with 10 mL cold fixation solution (methanol and acetic acid (3 : 1 *v*/*v*)) and stored at -20°C. Cells were resuspended in freshly made fixation solution and dropped on HistoBond+ slides (Marienfeld Superior). Slides were then stained with Giemsa and chromosomes were counted.

## 3. Results

### 3.1. Identification of a Chinese Peripheral Sclerocornea Pedigree Spanning Three Generations

**Figure 1:**
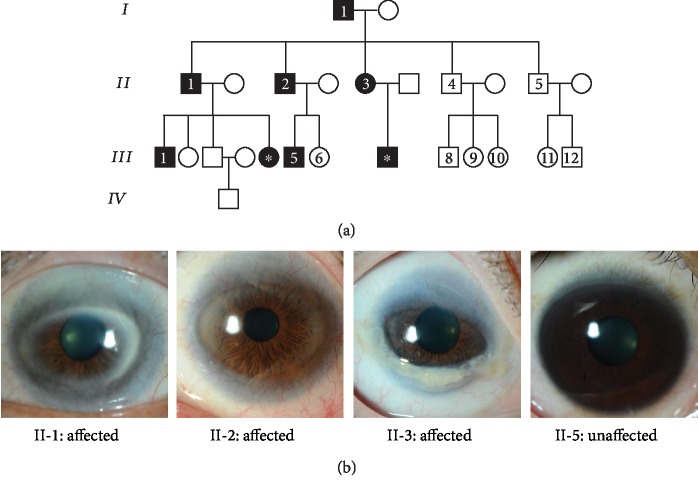
(a) The pedigree of a family showing autosomal dominant inherited peripheral sclerocornea. Black and white colors indicate affected and unaffected members, respectively, whereas squares and circles represent male and female, respectively. Roman numbers indicate the generations, and Arabic numbers show the family members who gave consent to clinical exams and genetic analysis. The closed square and circle carrying an asterisk in the third generation were identified to have peripheral sclerocornea solely based on histories. (b) Photographs of the eyes from three affected members (II-1, II-2, and II-3) and one unaffected individual (II-5). In the eyes of affected members, white regions were observed on the periphery of the corneas, indicating the peripheral sclerocornea. These white regions were not observed in the unaffected eye.

A nonconsanguineous Chinese family with peripheral sclerocornea was identified from the Hong Kong Eye Hospital. Eight cases (6 males and 2 females) of peripheral sclerocornea spanning across 3 generations were found in this family (Figure 1(a), black squares and circles). Fourteen members were examined with slit-lamp and 6 cases (I-1, II-1, II-2, II-3, III-1, and III-5) were identified to have peripheral sclerocornea (Figure 1(b)). Two additional cases in the third generation were identified to have peripheral sclerocornea solely based on histories. All affected subjects had bilateral sclerocornea. The degree of peripheral scleralization was similar in both eyes, despite the variations that existed among individuals. Peripheral sclerocornea was not noted in any of the examined unaffected subjects (II-4, II-5, III-6, III-8, III-9, III-10, III-11, and III-12). For all 14 members, the overall mean ± standard deviation and median age was 38.9 ± 21.6 years (range: 10-87 years) and 34.5 years, respectively. Comparing the affected and unaffected members, the mean age was 54.0 ± 20.6 years (range: 26-87 years; median 53.5 years) and 27.6 ± 14.8 years (range: 10-50 years; median 25.5 years), respectively (*p* = 0.020). In the affected subjects, two members (II-1 and II-3) had cataract and one member (I-1) had age-related macular degeneration.

### 3.2. Corneal Diameter but Not Central Corneal Opacity Was Influenced by Peripheral Cornea Scleralization

11 of the 14 members had best-corrected visual acuity (BCVA) of ≥20/30 in either eye. Three family members with peripheral sclerocornea had BCVA of <20/30. The mean logMAR BCVA was 0.17 ± 0.22 (range: 0–0.7) for all the 14 members of the family. Only myopia or mild hypermetropia was noted. The spherical equivalent ranged from -15.0 D to +2.5 D for all the 14 members. Comparing the affected and unaffected subjects, the mean spherical equivalent was −4.70 ± 5.97 diopters for the 6 affected members and −1.57 ± 2.89 diopters for the 7 unaffected members (*p* = 0.138). The mean horizontal and vertical corneal diameter for the affected subjects was 8.7 ± 1.4 mm and 6.6 ± 1.7 mm, respectively. The corresponding diameter was 11.6 ± 0.4 mm and 10.6 ± 0.5 mm for the unaffected subjects. Significant differences in both horizontal and vertical corneal diameter were observed between affected and unaffected family members (*p* = 0.013 and *p* = 0.001 for horizontal and vertical diameter, respectively).

**Figure 2:**
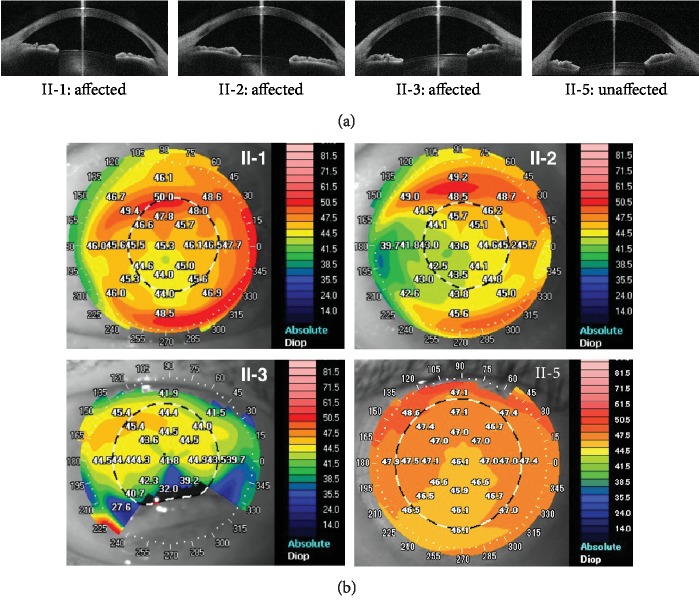
(a) Anterior segment imaging with the swept-source anterior segment OCT in selected family members (II-1, II-2, II-3, and II-5). Only horizontal section images of each subject are shown. No abnormality of the anterior chamber angle is found in affected and unaffected individuals. (b) Corneal topography of selected family members. Keratometry readings are shown on the corneas and represented by color codes as shown on the right. Top left: left eye image of II-1 (affected subject); top right: left eye image of II-2 (affected subject); bottom left: right eye image of II-3 (affected subject); bottom right: right eye image of II-5 (unaffected subject).

In all the 14 members, there were no corneal endothelial guttate, deep central corneal stromal opacities, iris abnormalities, iridocorneal adhesion, or shallow anterior chamber. Anterior chamber and angle analysis did not reveal any abnormality (Figure 2(a)). An open angle was identified in all cases. The intraocular pressure of both affected and unaffected subjects was comparable; the corresponding mean value was 14.6 ± 1.1 mmHg (range: 12 to 18 mmHg) and 16.3 ± 1.3 mmHg (range: 14 to 19 mmHg) (*p* = 0.073), respectively. There were no features of glaucoma either on optic disc assessment or retinal nerve fiber layer evaluation.

### 3.3. Mean Apex Pachymetry Differs Significantly between the Affected and Unaffected Members without Feature of Cornea Plana

The keratometric readings of the 6 affected subjects ranged from 41.9 to 46.5 diopters (mean: 44.1 ± 0.9 diopters), while the readings of the unaffected 7 subjects ranged from 42.7 to 46.2 diopters (mean: 44.8 ± 1.0 diopters) (Figure 2(b)) (*p* = 0.153). III-9 was excluded in this analysis because of the previous bilateral laser-assisted in situ keratomileusis (LASIK). The keratometry readings of affected subjects were within normal range and were not different from the unaffected subjects of the same family (Table 1). The mean preoperative keratometry for the affected subject who underwent bilateral cataract surgery (I-1) was 46.5 D for right eye and 44.5 D for left eye whereas the postoperative mean keratometry values were 47.3 D for right eye and 45.2 D for left eye. The mean apex pachymetry of the 6 affected members was 471 ± 39 *μ*m and the corresponding value of the unaffected members was 561 ± 19 *μ*m, which differed significantly (*p* = 0.003) (Table 1). The mean anterior chamber depths were 2.60 ± 0.60 mm and 3.11 ± 0.25 mm for the affected and unaffected members, respectively (*p* = 0.013) (Table 1). The anterior chamber depth of the affected eyes was shallower, although this may be explained by the older age of the affected family members. The anterior segment features in the affected members were unremarkable. Axial length of the affected subjects varied between 22.39 and 28.86 mm (mean: 24.67 ± 2.14 mm), while the axial length of the unaffected subjects ranged from 21.92 to 26.60 mm (mean: 24.29 ± 1.56 mm) (*p* = 0.796) (Table 1). None of the family members had mental deficits, metabolic syndromes, or systemic abnormalities except for hypertension in 3 cases. Serum lipid profile was normal for all 14 cases. The average keratometry and axial length in the affected subjects were not particularly hypermetropic as expected in cornea plana [21]. Therefore, we concluded that the affected members in our sclerocornea family do not show feature of cornea plana.

### 3.4. A Heterozygous Variant c.C1348T in *RAD21* Was Cosegregated with the Peripheral Sclerocornea in the Pedigree

**Figure 3:**
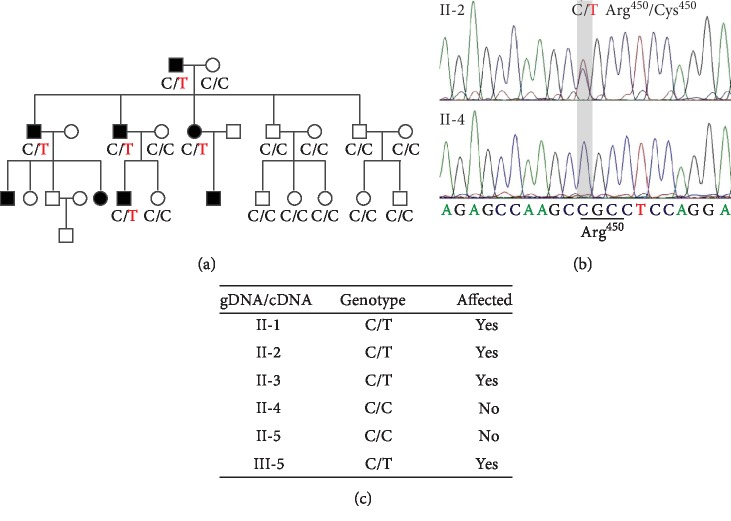
(a) C1348T of *RAD21* displayed complete cosegregation with peripheral sclerocornea, suggesting a strong association of C1348T *RAD21* with peripheral sclerocornea. Genotypes at 1348 position of RAD21 are shown under individuals. (b) Sanger sequencing spectra of *RAD21* alleles in affected and unaffected members. In the locus, the affected sample (II-2) showed two sequencing peaks, indicating both signals of dC and dT. The unaffected sample (II-4) showed only one peak of dC. (c) *RAD21* genotype of both genome DNA (gDNA) and transcripts (cDNA). The affected members carried both wild-type and variant *RAD21* alleles in their genome DNA and transcripts.

To identify genetic variants that are associated with sclerocornea in this family, we performed whole-exome sequencing (WES) in four affected (I-1, II-1, II-2, and II-3) and two unaffected (II-4 and II-5) members (Figure 1(a), Supplementary [Supplementary-material supplementary-material-1], Supplementary Tables [Supplementary-material supplementary-material-1], [Supplementary-material supplementary-material-1], and [Supplementary-material supplementary-material-1]). A total of 28,593 variants shared by the four affected members were analyzed using a stepwise filtering method, among which 3,744 variants caused protein sequence change. 2,838 of these coding variants can be found in the two unaffected WES results and were thus excluded. Given sclerocornea is a rare disease, a minor allele frequency threshold of 0.01% was applied to obtain 15 rare variants, including 8 nonsynonymous SNPs and 7 indels. We then validated these variants using the genome sequencing data of healthy Han Chinese from a recent myopia study [19]. This healthy group received comprehensive ophthalmological examinations, including visual acuity test, optical coherence tomography, and ocular imaging and showed no features of sclerocornea. Out of the 15 rare variants, 5 nonsynonymous SNPs were also found in the healthy Han Chinese group and were thus excluded for further analysis. In total, we identified 10 candidate variants associated with the peripheral sclerocornea phenotype (Supplementary [Supplementary-material supplementary-material-1]). Among these 10 variants, the missense variant *RAD21* c.C1348T (p.R450C) showed complete cosegregation with the peripheral sclerocornea in the pedigree (Figure 3(a)). This variant caused the change of arginine to cysteine at the amino acid position 450 of RAD21 and Arg450 is one of two separase cleavage sites. Sanger sequencing results confirmed that the affected members carry both wild-type and variants (Figure 3(b)).

### 3.5. RAD21 Variant Was Less Prone to Separase Mediated Cleavage but Caused No Change in Cell Cycles and Chromosome Segregation

**Figure 4:**
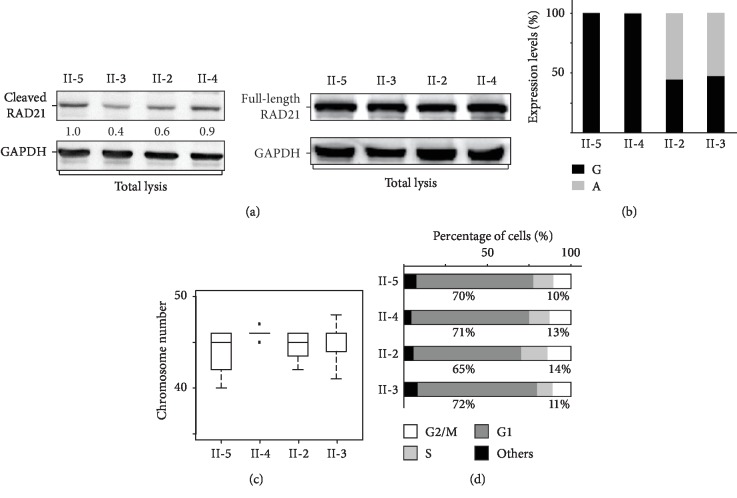
(a) Total protein extracts were analyzed using Western blotting. The intensities of the separase cleaved RAD21 bands decreased in affected members. Normalized levels of cleaved RAD21 relative to that of II-5 are shown under the Western blot image. Protein levels of the full-length RAD21 were comparable. GAPDH is shown as loading controls. (b) Similar expressions of wild-type and C1348T *RAD21* were detected in affected LCLs. (c) LCLs from affected members show normal ploidy. Metaphase chromosomes of each LCL sample were fixed and processed for chromosome counting. No significant difference in chromosome number was observed in LCLs isolated from affected and unaffected members. (d) Cell cycle profiles of each LCL. The affected LCLs did not show significant changes of cell cycle distribution.

Arg450 is one of the two separase cleavage sites on RAD21, we thus examined whether the R450C variant would affect the separase cleavage on RAD21. We first generated lymphoblastoid cell lines (LCLs) from peripheral blood of both affected (II-2 and II-3) and unaffected (II-4 and II-5) family members, respectively. We then genotyped both genomic DNA and cDNA to confirm these LCLs carry the *RAD21* c.C1348T allele in their genomic DNA and transcripts (Figure 3(c)). Our RNAseq results also confirmed that the RAD21 c.C1348T allele counted for 55% and 52% of the total *RAD21* expression levels in the affected member II-2 and II-3, respectively (Figure 4(b)), whereas unaffected LCLs only express the wild-type allele (Figure 4(b)). To examine whether the heterozygous RAD21 R450C variant could influence the separase cleavage on RAD21, we used a commercial antibody that can detect the 31.5 kDa protein fragment (amino acid 173-450) generated by separase cleavages on Arg172 and Arg450 of RAD21. Although the full-length protein levels of RAD21 show no difference between the affected and unaffected LCLs (Figure 4(a)), the RAD21^173-450^ protein levels were lower in the affected group than those in the unaffected group, suggesting less separase cleavage on the RAD21 C450 site (Figure 4(a)). It has been reported that mutations at both Arg172 and Arg450 on RAD21 impair sister chromosome segregation, which would lead to polyploidy [12]. We then counted chromosomes to examine the ploidy in LCLs. Our results showed that most LCLs have 46 chromosomes, the RAD21 R450C variant is thus insufficient to change the ploidy of the LCLs (Figure 4(c)). We also analysed the cell cycle profiles of LCLs and found no dramatic changes of cell cycle, particularly in G2/M phase (Figure 4(d)). In summary, these results showed that R450C decreases the cleavage efficiency of separase; however, reduced cleavage is insufficient to affect either ploidy or cell cycle.

## 4. Discussion

In this study, we identified a peripheral sclerocornea pedigree spanning over three generations. We performed systematic ophthalmic exams on 14 family members of this pedigree. The results showed that all affected members have nontransparent corneal rim and other features of peripheral sclerocornea. Although peripheral scleralization is often associated with cornea plana in the literature [22], the ophthalmic parameters of the affected members in this pedigree did not resemble that of cornea plana. All our clinical results indicated that peripheral sclerocornea of this pedigree is not complicated with other syndromes, thus allowing us to identify molecular causes for peripheral sclerocornea.

Although a few familial hereditary sclerocornea were reported [23, 24], sclerocornea-associated gene mutations have only been reported in individual sclerocornea patients. For example, heterozygous mutations in *RAX* were found in a single unilateral anophthalmia and sclerocornea case [25]. Mutations in *FOXE3* were reported to cause anterior segment ocular dysgenesis, including sclerocornea, microphthalmia, aphakia, and the absence of iris [26, 27]. Mutations at the chromosomal locus Xp22.3 were associated with an X chromosome-linked sclerocornea, microphthalmia, and dermal aplasia syndrome [28]. Although these mutations were reportedly associated with sclerocornea, clinical outcomes of these mutations are complicated with other syndromes. In this study, we identified a RAD21 R450C variant in a pedigree of peripheral sclerocornea. In addition to rs1051321465 (R450H), RAD21 R450C has been included as rs130282588 in the dbSNP database. However, neither population nor clinical association was reported with these two SNPs.

RAD21 contains two reported separase cleavage sites, and mutations of these two sites to alanine would lead to sister chromatids missegregation, cell cycle defects, and aneuploidy [12]. The variant identified in our pedigree is located on Arg450, one of the two separase cleavage sites, and our Western blotting result revealed less separase cleavage product on the RAD21 carrying this variant, suggesting R450C affects separase cleavage. However, wild-type RAD21 is still expressed in all affected members and the RAD21^R450C^ protein still possesses the other separase cleavage site R172, which could explain the apparently normal cell cycle and ploidy. If R172 is cleaved normally, a RAD21 protein fragment spanning from R172 to the stop codon is expected to be detected in affected members. However, we could not detect this fragment in Western blot, suggesting this fragment may be subjected to fast degeneration. Our results suggest that in addition to the well-studied cell cycle and chromosome segregation functions of RAD21, other novel RAD21 functions that could underline the pathogenesis of peripheral sclerocornea. Further studies are needed to clarify these novel functions of RAD21.

## Figures and Tables

**Table 1:** Ophthalmic examinations of a Chinese family with peripheral sclerocornea.

Affected cases	Age (years)	Eye	Average keratometry (diopter)	Average corneal diameter (mm)	Pachymetry (*μ*m)	Anterior chamber depth (mm)	Axial length (mm)
I-1	87	R	46.5	6.1	403	3.86^∗^	22.45
		L	44.5	5.8	409	3.88^∗^	22.39
II-1	63	R	43.4	6.5	472	2.01	23.08
		L	45.3	6.6	479	2.12	22.92
II-2	55	R	44.0	8.6	475	2.68	27.11
		L	43.6	8.5	476	2.60	24.36
II-3	52	R	41.9	6.7	521	2.49	23.62
		L	43.9	6.8	526	2.49	23.40
III-1	41	R	43.5	8.5	439	2.98	25.12
		L	43.2	8.4	428	2.92	25.32
III-5	26	R	44.3	9.8	486	2.84	27.49
		L	44.6	9.6	495	2.88	28.86
Average	54.0		44.1	7.7^∗∗^	471^∗∗^	2.60^∗∗^	24.67

Unaffected cases	Age (years)	Eye	Average keratometry (diopter)	Average corneal diameter (mm)	Pachymetry (*μ*m)	Anterior chamber depth (mm)	Axial length (mm)
II-4	50	R	45.1	10.8	578	2.88	23.12
		L	45.2	10.9	574	2.90	23.05
II-5	48	R	46.2	11.0	533	3.02	23.52
		L	45.7	11.1	550	3.05	23.68
III-6	23	R	45.0	11.2	565	2.96	26.06
		L	45.0	11.3	563	2.95	25.89
III-8	28	R	45.8	10.9	584	3.44	24.85
		L	45.6	10.9	590	3.45	24.70
III-9	27	R	38.0^∗^	11.6	408^∗^	3.22	25.50
		L	38.0^∗^	11.7	412^∗^	3.16	25.45
III-10	24	R	42.7	11.8	573	3.28	26.60
		L	42.9	11.8	574	3.30	26.07
III-11	11	R	44.8	10.8	550	2.77	22.53
		L	44.8	10.8	553	2.68	21.92
III-12	10	R	44.6	10.8	536	3.30	22.80
		L	44.2	10.9	533	3.37	22.88
Average	27.6		44.8	11.1^∗∗^	561^∗∗^	3.11^∗∗^	24.29

Mann–Whitney *U* test was used to compare the measurements between affected and unaffected subjects. An asterisk (^∗^) indicates the value excluded for statistical analysis because of the pseudophakic status in I-1 and previous bilateral laser-assisted in situ keratomileusis (LASIK) in III-9. Double asterisks (^∗∗^) indicate the presence of significant difference (*p* < 0.05) between affected and unaffected cases. R: right; L: left.

## Data Availability

The data used to support the findings of this study are included within the article.

## Abbreviations

BCVA: Best-corrected visual acuity
BWA: Burrows-Wheeler Aligner
CCO: Congenital corneal opacity
FBS: Fetal bovine serum
GATK: Genome Analysis Toolkit
IMDM: Iscove's modified Dulbecco's medium
LASIK: Laser-assisted in situ keratomileusis
LCLs: Lymphoblastoid cell lines
OCT: Optical coherence tomography
RNAseq: RNA sequencing
WES: Whole-exome sequencing.

## Supplementary Materials

Supplementary Materials Supplementary Figure 1: the analysis pipeline to process WES to identify sclerocornea-associated mutations from the pedigree. Supplementary Table 1: information of the 10 candidate variants resulting from filtering of exome sequencing data for the peripheral sclerocornea pedigree. Supplementary Table 2: summary of original exome sequencing data of the peripheral sclerocornea pedigree. Supplementary Table 3: summary of the detected variants of the peripheral sclerocornea pedigree.
