# Supplementary material for: A Cohesin Subunit Variant Identified from a Peripheral Sclerocornea Pedigree
Source: Dis Markers. 2019 Nov 12;2019:8781524. doi: 10.1155/2019/8781524 (PMC6875196; doi:10.1155/2019/8781524)
Supplement: Supplementary Materials — Supplementary Figure 1: the analysis pipeline to process WES to identify sclerocornea-associated mutations from the pedigree. Supplementary Table 1: information of the 10 candidate variants resulting from filtering of exome sequencing data for the peripheral sclerocornea pedigree. Supplementary Table 2: summary of original exome sequencing data of the peripheral sclerocornea pedigree. Supplementary Table 3: summary of the detected variants of the peripheral sclerocornea pedigree. [file 8781524.f1.pdf]

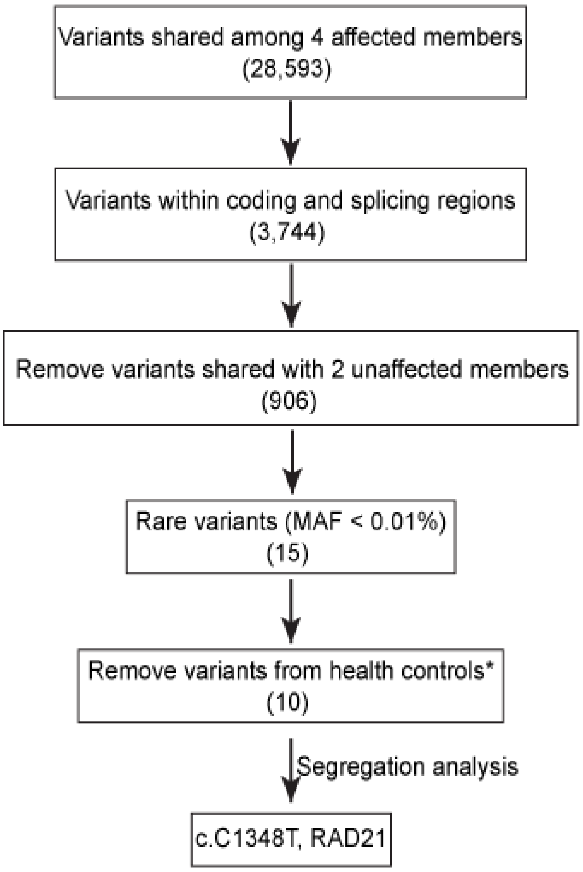

Supplementary Figure 1 The analysis pipeline to process WES to identify sclerocornea associated mutations from the pedigree. 3,744 variants within coding and splicing regions were obtained from 28,593 variants that are shared among 4 affected members. After removing the variants that are shared with 2 unaffected members, 906 variants were subjected to the next step. From these 906 variants, 15 rare variants were identified and among them, 5 were removed since they are shared among health individuals from a published cohort. After segregation analysis, c.C1348T in *RAD21* was chosen for studying its association with sclerocornea.

Supplementary Table 1 Information of the 10 candidate variants resulting from filtering of exome sequencing data for the peripheral sclerocornea pedigree.

No.	Gene Symbol	mRNA	cDNA change	dbSNP	Protein change	Clinical Significance	Reported Population
**1**	***RAD21***	**NM_006265**	**c.C1348T**	**rs1301282588**	**p. R450C**	**NA**	**NON**
2	*SQSTM1*	NM_001142299	c.C11T	rs763040103	p.S4F	NA	NON
		NM_003900	c.C263T		p.S88F		
		NM_001142298	c.C11T		p.S4F		
3	*RAPGEF5*	NM_012294	c.141_143del	NA	p.47_48del	NA	NA
4	*PRICKLE4*	NM_013397	c.863_864insTCT	rs76510495	p.L288delinsLL	NA	NA
5	*ANGPT2*	NM_001147	c.T704C	rs149699486	p.V235A	NA	NON
		NM_001118888	c.T548C		p.V183A		
		NM_001118887	c.T704C		p.V235A		
6	*MNT*	NM_020310	c.695_695+1insGT	rs58552657		NA	NA
7	*REXO1*	NM_020695	c.1741_1742insTCC	rs3052937	p.S581delinsSS	NA	NA
8	*OR7G3*	NM_001001958	c.922_923insATACC	rs3029651	p.I308fs	NA	AF*
9	*FAM98C*	NM_174905	c.1047_1048insAAG	rs59917662	p.K349delinsKK	NA	NON
10	*HSPBP1*	NM_012267	c.76_77insGGCGGCGGC	rs10701478	p.G26delinsGGGG	NA	NON
		NM_001130106	c.76_77insGGCGGCGGC		p.G26delinsGGGG		

NA: not available in dbSNP;
NON: non-association in dbSNP;
*AF: African 0.711, East Asian 0.365, Europe 0.314, South Asian 0.34, American 0.24.

Supplementary Table 2 Summary of original exome sequencing data of the peripheral sclerocornea pedigree.

**Data**	**I-1**	**II-1**	**II-2**	**II-3**	**II-4**	**II-5**
Number of raw reads (M)	58.9	92.5	80.0	62.2	77.0	70.0
Average read length (bp)	101	101	101	101	101	101
Raw data yield (Gb)	5.9	9.3	8.1	6.3	7.8	7.1
Number of reads mapped to the genome (M)	58.8	92.2	79.8	62.0	76.8	69.8
Fraction of uniquely mapped bases on target (%)	87.6%	87.5%	87.7%	87.7%	87.8%	87.2%
Data mapped to target region (M)	34.4	53.5	46.2	36.2	45.1	40.4
Mean depth of target region (fold)	44.7	69.6	60.2	47.0	58.8	52.4
Coverage of target region (%)	95.9%	96.1%	96.1%	95.8%	96.0%	96.1%
Target region with depth of more than 10 times (%)	90.0%	92.0%	91.7%	90.4%	91.4%	91.1%

Supplementary Table 3 Summary of detected variants of the peripheral sclerocornea pedigree.

**Variants**	**I-1**	**II-1**	**II-2**	**II-3**	**II-4**	**II-5**
Number of SNPs	76016	77937	77883	76800	77698	77649
Number of coding SNPs	20840	20572	20740	21087	20834	20928
Number of synonymous SNPs	10812	10719	10804	10981	10804	10866
Number of nonsynonymous SNPs	9475	9325	9402	9556	9483	9506
Number of Indels	7509	8170	8032	7610	7996	7754
Number of coding Indels	414	438	427	433	433	433
